# Association of dietary patterns with serum adipokines among Japanese: a cross-sectional study

**DOI:** 10.1186/s12937-015-0046-8

**Published:** 2015-06-11

**Authors:** Ikuko Kashino, Akiko Nanri, Kayo Kurotani, Shamima Akter, Kazuki Yasuda, Masao Sato, Hitomi Hayabuchi, Tetsuya Mizoue

**Affiliations:** 1 Department of Epidemiology and Prevention, Center for Clinical Sciences, National Center for Global Health and Medicine, Toyama 1-21-1, Shinjuku-ku, Tokyo, 162-8655 Japan; 2 Department of Metabolic Disorder, Diabetes Research Center, National Center for Global Health and Medicine, Tokyo, Japan; 3 Department of Applied Biological Chemistry, Graduate School of Bioresource and Bioenvironmental Sciences, Kyushu University, Fukuoka, Japan; 4 Graduate School of Nutrition and Health Science, Fukuoka Women’s University, Fukuoka, Japan

**Keywords:** Cross-sectional study, Dietary pattern, Adipokines, Adiponectin, Leptin, PAI-1, Japanese, Epidemiology

## Abstract

**Background:**

Diet may influence disease risk by modulating adipokines. Although some foods and nutrients have been linked to circulating adipokine levels, little is known about the role of dietary patterns on adipokines. We investigated the association between major dietary patterns and circulating levels of adiponectin, leptin, resistin, visfatin, and plasminogen activator inhibitor-1 (PAI-1) in a working population.

**Methods:**

The subjects were 509 employees (296 men and 213 women), aged 20 to 65 years, of two municipal offices. Serum adipokines were measured using a Luminex suspension bead-based multiplexed array. Dietary patterns were derived by using principal component analysis of the consumption of 52 food and beverage items, which were ascertained by a validated diet history questionnaire. Multiple regression analysis was performed to assess the association between dietary pattern scores and adipokine concentrations, with adjustment for potential confounders.

**Results:**

Three major dietary patterns were extracted: a Japanese, a Westernized breakfast, and a meat food patterns. Of these, we found significant, inverse associations of the Westernized breakfast pattern, which was characterized by higher intake of confectioneries, bread, and milk and yogurt but lower intake of alcoholic beverages and rice, with serum leptin and PAI-1 concentrations in a fully adjusted model (*P* for trend = 0.04 for both leptin and PAI-1). The other adipokines were not significantly associated with any dietary pattern.

**Conclusion:**

The Westernized breakfast dietary pattern may be associated with lower circulating levels of leptin and PAI-1.

## Introduction

Adipokines such as adiponectin, leptin, resistin, visfatin, and plasminogen activator inhibitor type-1 (PAI-1) are secreted from adipose tissues. Adiponectin has been positively associated with insulin sensitivity [1] and lowered risk of diabetes [2], hypertension [3], and coronary heart disease (CHD) [4]. In contrast, higher blood leptin levels have been associated with a higher risk of type 2 diabetes [5], hypertension [6], and CHD [7]. Other adipokines such as resistin, visfatin, and PAI-1 have been identified as pro-inflammatory mediators, and are related to insulin resistance and type 2 diabetes [8–10]. As adipokines are presumed to be biochemical predictors of these diseases [2–9], it is important to identify modifiable factors including diet that may affect adipokines.

Previous studies have demonstrated a correlation between nutrients such omega-3 fatty acid [11] and dietary fiber [12, 13] and foods or food groups such as vegetables [12, 14], fish [15], soybean [12], sweetened beverages [16], and coffee [17] and blood adipokine levels. Given that nutrients and foods are consumed in combination, studies that seek to take account of potential interactions or synergistic effects among nutrients and foods need to evaluate the diet as a whole. For instance, the Mediterranean diet and a healthy eating pattern in the U.S. were associated with higher concentrations of adiponectin [18–20] and lower concentrations of resistin [20], while a Chinese study reported that the Western pattern characterized by higher intake of milk, meats, cake, and fruits was positively related to serum leptin levels [21]. In contrast, other studies in the U.S. showed no association between Western pattern and leptin levels [22, 23]. The lower age-adjusted mortality rate of cardiovascular disease (CVD) in Japan compared with Western countries such as the U.K. and U.S. [24] might be ascribable to the favorable effect of the Japanese diet, which is rich in fish, seaweeds, soybean, and vegetables [25, 26]. It would thus be interesting to determine whether distinctive dietary patterns for Japanese influence circulating adipokine levels, but to our knowledge only one study has examined the relationship between dietary pattern and blood adiponectin level among Japanese [27].

Here, we examined the association between major dietary patterns and serum adipokine levels in a Japanese working population by utilizing dietary pattern analysis.

## Methods

### Study procedure and subjects

A health survey was conducted during a periodic health examination among employees of two municipal offices in northeastern Kyushu, Japan (conducted in July 2009 in one office and November 2009 in another), as described elsewhere [17]. In brief, all full-time workers (n = 605) were invited to participate in the survey and asked to fill out the questionnaires. Of 605 eligible employees, 567 (325 men and 242 women) participated in the survey (response rate 94 %). Of these, we excluded 23 subjects with a history of CVD (n = 11) and cancer (n = 13) from analysis of dietary patterns, and a further 20 subjects with a history of diabetes (n = 8), nephritis (n = 1), chronic hepatitis (n = 3), as well as pregnant women (n = 8). We also excluded subjects who had missing data on serum adipokines (n = 20) and any of the covariates (n = 6). Some of the excluded subjects had two or more conditions for exclusion. Finally, 509 subjects (296 men and 213 women) aged 20 to 65 years were analysed for the present study. Additionally, we analysed 508 subjects in the analysis of visfatin after excluding one subject with a visfatin concentration above the upper detection limit. With regard to leptin, analyses were performed among those subjects who participated in the checkup in the fasting state (at least 8 h) since the last meal (n = 486). The protocol of the study was approved by the Ethics Committee of the National Center for Global Health and Medicine, and written informed consent was obtained from each participant.

### Measurement of adipokines

Blood samples were obtained on the day of the health checkup. Serum samples for measurement of adipokines were stored at -80 °C until biochemical assay. Serum concentrations of adiponectin, leptin, resistin, visfatin, and PAI-1 were measured using a Luminex suspension bead-based multiplexed array using a Bio-Plex 3D suspension array system and Bio-Plex Pro human diabetes assay panel (Bio-Rad Laboratories, Hercules, CA); intra-assay coefficients of variation were 12 % for adiponectin, 11 % for leptin, 8 % for resistin, 19 % for visfatin, and 21 % for PAI-1 [17].

### Dietary assessment

Dietary habits during the preceding month were evaluated using a validated brief self-administered diet history questionnaire (BDHQ), as described elsewhere [28]. In brief, dietary intakes for 58 food and beverage items commonly consumed in Japan were estimated using an ad hoc computer algorithm [29]. According to a validation study of BDHQ using 16-day semi-weight dietary records as gold standard, median Spearman’s correlation coefficients (range) for 58 food and beverages items were 0.44 (0.14-0.82) in women and 0.48 (0.22-0.83) in men [28].

### Other variables

The survey questionnaire also enquired about marital status, smoking status, type of occupation, and non-occupational physical activity. Occupational physical activity was classified as sedentary work and active work. Non-occupational physical activity was evaluated from the number of minutes per day spent in walking or cycling during commuting to or from work and the number of hours per week spent in each of five leisure activities (walking, low-, moderate-, and high-intensity activities, and gardening). Non-occupational physical activities were estimated from the metabolic equivalent (MET) value and expressed as the sum of METs multiplied by the time (in hours) spent performing each activity. Body height was measured to the nearest 0.1 cm with subjects standing without shoes. Body weight in light clothes was measured to the nearest 0.1 kg. Body mass index (BMI) was calculated by dividing weight by squared height (kg/m^2^).

### Statistical analysis

We performed principal component analysis on the basis of energy-adjusted intakes using the density method (amount of food intake per 1000 kcal of energy) for 52 food and non-alcoholic or alcoholic beverage items, excluding six items (sugar added to coffee or black tea, salt, oil, sugar, table salt and salt-containing seasoning added at the table, and soup consumed with noodles). The factors were rotated by orthogonal transformation (varimax rotation) to maintain uncorrelated factors and greater interpretability. We determined the number of factors to retain by considering eigenvalues, the scree test, and the interpretability of factors. Three dietary patterns were identified. Dietary patterns were named according to the food items showing a high load (absolute value) in three factors. The factor scores for each dietary pattern and for each individual were calculated by summing the intakes of food items weighted by their factor loading. Factor scores were categorized into tertiles.

Participants were classified into tertiles of dietary pattern. Subject characteristics were expressed as means (standard deviation) for continuous variables and percentages for categorical variables. We examined the associations between dietary patterns and serum adipokine concentrations by multiple regression analysis after first log-transforming the adipokine concentrations because of their highly skewed distributions. The results were presented as geometric means and their 95 % confidence interval (CI). In model 1, we adjusted for age (years, continuous), sex, and workplace (site A or B). In model 2, we additionally adjusted for marital status (married or not), BMI (kg/m^2^, continuous), occupational physical activity (sedentary or active work), non-occupational physical activity (0, 0 < - < 5 MET-hr/week, 5- < 10 MET-hr/week, ≥10 MET-hr/week), smoking (never smokers, ex-smokers, current smokers consuming 1–19 cigarettes/day, or current smokers consuming ≥20 cigarettes/day), and total energy intake (kcal/day, continuous). The trend association was tested in each model, with ordinal numbers 0–2 assigned to the tertile categories of each dietary pattern. In addition, we performed stratified analyses of dietary patterns and serum leptin and PAI-1 according to sex and BMI (<23 or ≥23 kg/m^2^). To assess statistical interactions, we created interaction terms by multiplying dietary patterns (tertile) and the above stratifying variables (dichotomous) and added these to the models. Two-sided *P* values <0.05 were regarded as statistically significant. All analyses were performed using the SAS statistical software package version 9.3 (SAS Institute Inc., Cary, NC, USA).

## Results

We identified three major dietary patterns by principal component analysis (Table 1). The first factor was named a Japanese pattern. This pattern was characterized by vegetables, fruits, mushrooms, seaweeds, and soy products. The second factor was named a Westernized breakfast pattern, because it had higher scores for confectioneries, bread, and milk and yogurt but lower scores for alcoholic beverages and rice. The third factor was named a meat food pattern because it represented higher intakes of meat, processed meat, and mayonnaise and dressing. The first to third dietary patterns accounted for 10.3 %, 5.4 %, and 4.3 %, respectively, of the variance in food intake and in total explained 20.0 % of variability.

**Table 1:** Factor loading matrix for dietary patterns identified by principal component analysis^a^

	Japanese pattern	Westernized breakfast pattern	Meat food pattern
Cabbage/Chinese cabbage	0.75	-	-
Carrots/pumpkin	0.74	-	-
Green leaves vegetables	0.72	-	-
Mushrooms	0.70	−0.15	-
Other root vegetables	0.69	-	-
Japanese radish/turnip	0.65	-	−0.19
Lettuces/cabbage (raw)	0.55	-	0.20
Seaweeds	0.53	−0.22	−0.18
Potatoes	0.45	-	-
Tomatoes	0.42	-	0.15
Tofu/atsuage^b^	0.35	−0.27	−0.24
Persimmons/strawberries/kiwifruit	0.33	0.17	−0.31
Other fruit	0.31	0.22	−0.24
Pickled green leaves vegetables	0.24	−0.20	−0.23
Lean fish	0.22	−0.18	0.15
Natto^c^	0.20	-	−0.16
Green tea	0.20	-	-
Sake	−0.15	-	-
Whisky	−0.17	-	-
Buckwheat noodles	−0.18	−0.15	-
Japanese wheat noodles	−0.18	-	-
Cola drink/soft drink	−0.22	0.17	-
Chinese noodles	−0.34	−0.20	-
Western-type confectioneries	-	0.64	-
Japanese confectioneries	0.15	0.55	−0.19
Rice crackers/rice cake/okonomiyaki^d^	-	0.50	-
Bread	-	0.46	0.24
Milk and yogurt	-	0.33	0.16
Ice cream	−0.23	0.28	-
100 % fruit and vegetable juice	-	0.17	-
Coffee	-	0.17	-
Oily fish	-	−0.21	-
Other pickles	0.18	−0.25	−0.16
Dried fish/salted fish	-	−0.25	-
Liver	-	−0.27	-
Beer	−0.21	−0.30	0.15
Shochu	−0.24	−0.39	-
Pork/beef	-	-	0.49
Chicken	-	−0.15	0.46
Ham/sausage/bacon	-	-	0.38
Mayonnaise/dressing	0.24	-	0.37
Black tea/oolong tea	-	0.15	0.29
Egg	-	-	0.26
Spaghetti and macaroni	-	-	0.26
Squid/octopus/shrimp/shellfish	-	−0.21	0.23
Canned tuna	-	-	0.20
Low-fat milk and yogurt	-	-	−0.16
Small fish with bones	0.18	−0.22	−0.25
Miso soup	-	−0.24	−0.36
Citrus fruit	0.29	0.19	−0.36
Rice	−0.34	−0.28	−0.48

^a^Factor loading less than ±0.15 is represented by a dash for simplicity. The table omits food items with factor loadings less than ±0.15 for all dietary patterns (wine)

^b^Deep-fried tofu

^c^Fermented soybeans

^d^Meat/fish and vegetable pancake

Table 2 shows the characteristics of the study subjects according to tertile categories of each dietary pattern score. Subjects with a higher score for the Japanese pattern were more likely to be female and have a lower BMI, but were less likely to be smokers and engaged in sedentary work. Subjects with a higher score for the Westernized breakfast pattern were younger and more likely to be female and physically active during work, and have a lower BMI, but were less likely to be married and smokers, and tended to have lower energy intake. Regarding the meat food pattern, subjects with a higher score were younger.

**Table 2:** Subject characteristics by dietary pattern (n = 509)

	Japanese pattern			Westernized breakfast pattern			Meat food pattern		
	T1 (low)	T2	T3 (high)	T1 (low)	T2	T3 (high)	T1 (low)	T2	T3 (high)
No. of subjects	169	170	170	170	170	169	169	170	170
Age (years), mean ± SD	43.4 ± 10.9	43.5 ± 11.1	43.4 ± 10.8	46.6 ± 11.7	42.5 ± 10.1	41.2 ± 10.4	47.9 ± 10.9	43.2 ± 10.9	39.2 ± 9.3
Men (%)	84.6	58.2	31.8	74.6	60.6	39.4	58.6	63.5	52.4
Workplace (site A) (%)	32.5	31.8	24.1	31.4	25.3	32.0	18.3	34.1	35.9
Married (%)	74.0	72.9	70.6	80.5	70.0	67.1	75.7	72.9	68.8
BMI (kg/m^2^), mean ± SD	22.9 ± 3.1	22.3 ± 3.3	21.8 ± 3.5	22.9 ± 3.3	22.4 ± 3.4	21.7 ± 3.2	22.3 ± 3.2	22.6 ± 3.4	22.1 ± 3.3
Sedentary work (%)	87.6	80.6	75.3	84.7	81.8	77.1	76.9	82.4	84.1
Non-job physical activity (≥5 MET-hr/week) (%)	34.9	37.1	35.3	42.6	37.1	27.7	39.1	35.3	32.9
Current smoker (%)	39.1	24.1	12.4	38.5	24.1	12.9	24.9	26.5	24.1
Total energy intake (kcal/day)	1827 ± 503	1752 ± 529	1773 ± 491	1890 ± 539	1786 ± 526	1676 ± 433	1747 ± 522	1861 ± 517	1743 ± 478

*T1 to T3* tertiles 1 to 3, *BMI* body mass index

As shown in Table 3, leptin and PAI-I concentrations decreased with increasing scores of the Westernized breakfast pattern. In the multivariable-adjusted model (model 2), the adjusted geometric means of leptin concentration for the lowest through highest tertiles of the Westernized breakfast pattern score were 1.82, 1.57, and 1.54 ng/ml, respectively (*P* for trend = 0.04), while those of PAI-1 were 31.87, 30.46, and 29.68 ng/ml, respectively (*P* for trend = 0.04). The Japanese and meat food patterns were not significantly associated with any adipokine; however, those in the middle and highest tertiles tended to have higher adiponectin levels than those in the lowest tertile (geometric mean: 4.89 and 4.85 versus 4.44 μg/ml).

**Table 3:** Geometric means (95 % CI) of serum adipokine concentrations by tertile of dietary pattern score

	No. of subjects	Tertile categories of dietary pattern scores			
		T1 (low)	T2	T3 (high)	Trend *P*^a^
*Adiponectin (μg/ml)*					
Japanese pattern					
Model 1	509	4.46 (4.00–4.98)	4.91 (4.45–5.43)	4.84 (4.36–5.37)	0.30
Model 2	509	4.44 (3.89–5.08)	4.89 (4.31–5.54)	4.85 (4.26–5.53)	0.26
Westernized breakfast pattern					
Model 1	509	4.64 (4.18–5.16)	4.70 (4.24–5.21)	4.87 (4.41–5.39)	0.51
Model 2	509	4.68 (4.12–5.31)	4.71 (4.13–5.36)	4.83 (4.23–5.51)	0.69
Meat food pattern					
Model 1	509	4.88 (4.38–5.44)	4.74 (4.28–5.23)	4.64 (4.20–5.13)	0.50
Model 2	509	4.80 (4.20–5.49)	4.73 (4.17–5.36)	4.68 (4.12–5.33)	0.75
*Leptin (ng/ml)*					
Japanese pattern					
Model 1	486	1.70 (1.47–1.97)	1.68 (1.46–1.93)	1.76 (1.53–2.03)	0.72
Model 2	486	1.64 (1.42–1.90)	1.64 (1.42–1.88)	1.67 (1.45–1.93)	0.85
Westernized breakfast pattern					
Model 1	486	1.88 (1.63–2.17)	1.65 (1.44–1.90)	1.64 (1.43–1.88)	0.17
Model 2	486	1.82 (1.58–2.09)	1.57 (1.37–1.81)	1.54 (1.33–1.78)	0.04
Meat food pattern					
Model 1	486	1.61 (1.39–1.87)	1.78 (1.56–2.04)	1.74 (1.51–2.00)	0.48
Model 2	486	1.66 (1.44–1.93)	1.68 (1.46–1.92)	1.61 (1.39–1.86)	0.68
*Resistin (ng/ml)*					
Japanese pattern					
Model 1	509	3.43 (3.14–3.75)	3.39 (3.12–3.68)	3.13 (2.88–3.41)	0.15
Model 2	509	3.59 (3.21–4.00)	3.59 (3.23–3.98)	3.32 (2.98–3.70)	0.24
Westernized breakfast pattern					
Model 1	509	3.32 (3.04–3.62)	3.20 (2.94–3.48)	3.40 (3.13–3.69)	0.67
Model 2	509	3.48 (3.14–3.87)	3.37 (3.03–3.76)	3.63 (3.25–4.05)	0.51
Meat food pattern					
Model 1	509	3.45 (3.16–3.77)	3.32 (3.06–3.60)	3.18 (2.93–3.46)	0.18
Model 2	509	3.64 (3.26–4.07)	3.50 (3.16–3.89)	3.36 (3.02–3.74)	0.20
*Visfatin (ng/ml)*					
Japanese pattern					
Model 1	508	0.86 (0.74–1.01)	0.84 (0.73–0.97)	1.04 (0.89–1.20)	0.10
Model 2	508	0.93 (0.77–1.13)	0.91 (0.75–1.09)	1.12 (0.92–1.36)	0.11
Westernized breakfast pattern					
Model 1	508	0.92 (0.80–1.07)	1.00 (0.86–1.15)	0.84 (0.73–0.97)	0.35
Model 2	508	0.98 (0.81–1.18)	1.07 (0.89–1.30)	0.91 (0.75–1.10)	0.49
Meat food pattern					
Model 1	508	0.85 (0.73–0.99)	0.93 (0.81–1.07)	0.95 (0.82–1.10)	0.30
Model 2	508	0.92 (0.76–1.12)	0.99 (0.83–1.20)	1.02 (0.85–1.23)	0.36
*PAI-1 (ng/ml)*					
Japanese pattern					
Model 1	509	30.41 (28.92–31.98)	29.50 (28.17–30.89)	28.71 (27.37–30.12)	0.11
Model 2	509	31.48 (29.60–33.47)	30.73 (29.00–32.57)	30.08 (28.32–31.96)	0.21
Westernized breakfast pattern					
Model 1	509	30.96 (29.50–32.50)	29.27 (27.92–30.68)	28.45 (27.17–29.80)	0.01
Model 2	509	31.87 (30.07–33.78)	30.46 (28.70–32.33)	29.68 (27.93–31.54)	0.04
Meat food pattern					
Model 1	509	29.81 (28.36–31.33)	29.75 (28.40–31.16)	28.99 (27.67–30.38)	0.42
Model 2	509	31.42 (29.54–33.41)	31.03 (29.29–32.89)	29.88 (28.16–31.71)	0.15

*CI* confidence interval, *PAI-1* plasminogen activator inhibitor-1

^a^Based on multiple regression analysis, with ordinal numbers 0 to 2 assigned to the tertile categories of each dietary pattern

Model 1: adjusted for age (years, continuous), sex, workplace (site A or B)

Model 2: additionally adjusted for married (yes or no), body mass index (kg/m^2^, continuous), occupational physical activity (sedentary and active), non-occupational physical activity (0, 0 < - < 5 MET-hr/week, 5- < 10 MET-hr/week, or ≥10 MET-hr/week), smoking (never smokers, ex-smokers, current smokers consuming 1–19 cigarettes/day, or current smokers consuming ≥20 cigarettes/day), and total energy intake (kcal/day, continuous)

In stratified analyses, the inverse associations between the Westernized breakfast pattern and serum leptin and PAI-1 concentrations were confined to men (*P* for trend = 0.002 for leptin and 0.01 for PAI-1) and to overweight subjects (*P* for trend = 0.02 for leptin and 0.05 for PAI-1), although the interaction by sex or BMI was not statistically significant (*P* for interaction by sex = 0.19 for leptin and 0.33 for PAI-1, *P* for interaction by BMI = 0.50 for leptin and 0.47 for PAI-1).

## Discussion

In this study in Japanese workers, we identified three major dietary patterns: a Japanese, a Westernized breakfast, and a meat food dietary pattern. Of these, the Westernized breakfast pattern, characterized by higher intake of confectioneries, bread, and milk and yogurt but lower intake of alcoholic beverages and rice, was significantly inversely associated with serum leptin and PAI-1 concentrations after adjustment for potential confounding factors, including BMI. These inverse associations were evident in men and obese subjects. The other adipokines were not significantly associated with any dietary pattern. To our knowledge, this study is one of only a few studies to examine the associations between dietary patterns and adipokines in an Asian population.

The dietary patterns identified in the present study were similar to those in previous Japanese and Western studies: a Japanese pattern (similar to the Japanese pattern in previous studies [25, 27, 30, 31]); a Westernized breakfast pattern (similar to the sweets-fruits pattern [27, 30–32]); and a meat food pattern (similar to the Western pattern [21–23, 25]). Of these patterns, the Westernized breakfast pattern showed significant inverse associations with leptin and PAI-1 levels. We are not aware of any study that has reported associations between similar dietary patterns and these adipokines. This finding might be explained by some foods and nutrients that contributed to the Westernized breakfast pattern. With regard to dairy food, a prospective case-control study reported a significant inverse correlation between the sum of pentadecanoic acid and heptadecanoic acid in serum phospholipids (biomarkers of milk fat intake) and leptin and PAI-1 [33]. Conjugated linoleic acid, which is rich in dairy products, was inversely associated with PAI-1 in obese rats [34]. In the present study population, higher consumption of coffee, which modestly contributed to the Westernized breakfast pattern (factor loading score = 0.17), was associated with lower levels of leptin and PAI-1 [17]. The present finding is also compatible with those of our previous studies in the same setting that examined associations between dietary pattern and other related outcomes. Specifically, scores of dietary patterns characterized by the frequent intake of bread and dairy products but infrequent intake of alcohol, similar to the present Westernized breakfast pattern, have been inversely associated with the prevalence of metabolic syndrome [30], high blood pressure [30], and glucose tolerance abnormalities [32] and lower concentrations of C-peptide [31]. These abnormalities have also been linked to leptin [6, 35, 36] and PAI-I levels [37]. Therefore, the Westernized breakfast pattern may decrease risks of these metabolic diseases by lowering circulating leptin and PAI-1levels.

Guo et al. reported that a Japanese dietary pattern, which was characterized by a higher intake of vegetables, seaweeds, tofu, and fish, was significantly associated with higher serum adiponectin concentrations in men [27]. In our study, serum adiponectin levels in the middle and highest tertiles of the Japanese pattern, characterized by vegetables, fruits, mushrooms, seaweeds, and soy products, tended to be higher than those in the lowest level of this pattern, although the trend association was not statistically significant. The lack of a significant association in the present population may be ascribed to the lower factor loading scores of fish and shellfish and soy products compared to those of the previous study (fish and shellfish, 0.47 in the previous study vs 0.02 to 0.22 in our study; soy products, 0.31 to 0.60 in the previous study vs -0.03 to 0.35 in our study) [27]. A review of studies in humans concluded that a fish-based diet and an omega-3 fatty acid-rich diet may increase circulating adiponectin levels [11]. Additionally, rats fed with soy protein diet showed higher plasma adiponectin concentrations than rats fed with a casein diet [38]. A dietary pattern with higher loadings of both fish and soy foods may show a clearer association with adiponectin concentrations.

We found no association between the meat food pattern, characterized by higher intake of meat, processed meat, and mayonnaise and dressing, and serum levels of adipokines, including leptin. A few previous studies reported an association between meat food patterns and leptin. A study in the U.S. found no association between a pattern characterized by higher intakes of red meats, processed meats, high-energy drinks, and refined grains and serum leptin [23]. Another U.S. study showed that a dietary pattern characterized by higher intakes of red meats, processed meats, french fries, and eggs was positively related to leptin levels, but that the association lost statistical significance after additional adjustment for BMI [22]. In contrast, a dietary pattern characterized by higher intake of milk, meats, cake, and fruits was positively related to leptin levels in Chinese men and women [21]. The majority of studies to date do not support an independent role of diet with a high meat intake in the regulation of circulating leptin.

Our study has a number of strengths, including its high response rate (94 %), use of a validated dietary questionnaire, and adjustment for potentially important confounding factors. This study has also several limitations. First, dietary patterns extracted based on principal component analysis involves subjective decisions in determining the number of factors to retain, in choosing the method of rotation of the initial factors, and in labeling the dietary patterns. However, we identified major dietary patterns that have previously been reported in Japanese as well as Western populations. Second, some food items demonstrated relatively low validities. For instance, Spearman’s rank correlation coefficients for seaweeds and potatoes were less than 0.2 in men. This might partly account for the lack of clear associations for the Japanese dietary pattern. Third, we measured adipokine levels in serum that was obtained at a single point in time, and the measured data may not be adequately reflect long term status. Nevertheless, plasma adipokine levels have been shown to be relatively stable across season [39]. Fourth, because the present study was cross-sectional, we were unable to conclude that the observed associations were causal. To minimize the possibility of reverse causality, however, we excluded subjects with chronic diseases that might affect dietary habit and adipokines, including cardiovascular disease, cancer, and diabetes. Fifth, our sample size was relatively small (n = 509), which lack sufficient power to detect moderate associations with statistical significance. Finally, the present findings were derived from only employees of two municipal offices in northeastern Kyushu, Japan, which may not be the representative of general Japanese population.

## Conclusion

We found that a Westernized breakfast pattern was inversely related to serum leptin and PAI-1 concentrations. This cross-sectional observation awaits prospective confirmation.

## Abbreviations

BDHQ: Brief self-administered diet history questionnaire
BMI: Body mass index
CHD: Coronary heart disease
CI: Confidence interval
CVD: Cardiovascular disease
MET: Metabolic equivalent
PAI-1: Plasminogen activator inhibitor type-1
